# Simulated data from a genotype-to-phenotype crop growth model for pepper

**DOI:** 10.1016/j.dib.2021.107119

**Published:** 2021-05-11

**Authors:** Paulo Canas Rodrigues

**Affiliations:** Federal University of Bahia, Brazil

**Keywords:** Plant sciences, Genotype-by-environment interaction, Crop growth model, Genotype-to-phenotype simulation model, Quantitative genetics

## Abstract

The data in this article includes 300 simulated two-way data tables with 200 genotypes in the rows and 12 environments in the columns each. The yield data was obtained from a genotype-to-phenotype crop growth model that was adapted for pepper. The genotypes were characterized by 237 markers covering all the 12 chromosomes, and the environments were obtained as a combination of: (i) two levels of radiation based on historical data; (ii) three levels of daily average temperatures, 15, 20 and 25 °C; and (iii) two countries, Spain and The Netherlands. 100 two-way data tables were obtained for each of the three levels of heritability in the environments, 0.3, 0.5 and 0.8. The data is available as supplementary material of this paper.

## Specifications Table

**Table:** 

Subject	Agronomy and Crop Science
Specific subject area	Plant breeding
Type of data	Table Spreadsheet
How data were acquired	The data was obtained based on a genotype-to-phenotype crop growth simulation model. The R script to generate the data is available in Rodrigues [5] and the supplementary material, together with two cvd files needed to run the code.
Data format	Raw
Parameters for data collection	Two levels of radiation based on historical data (minimum and maximum) for both Spain and The Netherlands were considered, together with three levels of daily average temperatures, 15, 20 and 25 °C.
Description of data collection	The data was obtained based on a genotype-to-phenotype crop growth simulation model
Data source location	Simulation model, created while visiting the Wageningen University, The Netherlands.
Data accessibility	With the article As supplementary material https://data.mendeley.com/datasets/76sjsc5j7v/2, doi:10.17632/76sjsc5j7v.2
Related research article	P.C. Rodrigues, E. Heuvelink, L.F.M. Marcelis, S. Chapman, F. van Eeuwijk, An analysis of synthetic yield data for pepper shows how genotype by environment interaction in yield can be understood in terms of yield components and their QTLs. Crop Science, 2021. DOI:10.1002/csc2.20476

## Value of the Data

• The data (300 data tables) will be useful to validate and compare statistical methods for modelling genotype-by-environment interaction
• Researchers and practitioners can in plant science and agronomy can benefit from these data
• The data can be used for general model comparison and simulation in the context of plant sciences and in the context of genotype-by-environment studies
• The data can be used to compare methods for stability analysis

## 1 Data Description

Two-way data tables to structure and understand genotype-by-environment interactions in plant sciences are of key importance to improve genotype adaptability and maximize yield [4], [6]. This data article presents 300 simulated two-way data tables of yields with 200 genotypes in the rows and 12 environments in the columns, 100 for each level of heritability in the environments, 0.3, 0.5 and 0.8 (Supplementary Material). The yield data was obtained from a genotype-to-phenotype crop growth model, adapted for pepper. The genotypes were characterized by 237 markers covering all the 12 chromosomes, following Barchi et al. [1]. The environments were obtained as a combination of: (i) two levels of radiation based on historical data; (ii) three levels of daily average temperatures, 15, 20 and 25 °C; and (iii) two countries, Spain and The Netherlands. The codes in the data files are of the form “XXa-bb”, with XX being NL for The Netherlands and SP for Spain, a=1,5 for lower and higher radiation in the historical data, and bb=15,20,25 for the respective temperature. The R script to generate the data is available in the supplementary material, together with two csv files needed to run the code.

## 2 Experimental Design, Materials and Methods

A physiological genotype-to-phenotype model was used in which only growth-defining factors determine the maximum production that can be achieved under given environmental conditions and crop characteristics. The model under study was developed by Rodrigues [8] and by Rodrigues et al. [3], and applied by Rodrigues et al. [7], and allows to write the yield of genotype i in environment j as

(1) $$Yield_{i,j}=\;\frac{FTF_{i}\times \;\left[1-W_{i}\times \left(T_{j}-T_{FTF}\right)\right]}{FDMC_{i}}\times LUE_{i,j}\times \sum_{t=t_{0}}^{t_{f}}\left[1-\exp(-K_{i}\times LAI_{i,j,t})\right]\times I_{j,t},$$

where FDMCi is the fruit dry matter content of genotype i, $T_{j}$ defines the l levels of daily average temperature, constant across all growing season, t0 and tf are the indices of the first and last days of the growing season, K is the light extinction coefficient, and the function $\left\{1-W_{i}\times \left(T_{j}-T_{FTF}\right)\right\}$ represents a genotypic-specific linear reduction in fraction partitioned to the fruits (FTF) for temperatures above TFTF = 15 °C. The light use efficiency (LUE) can be written as

(2) $$LUE_{i,j}=LUE_{i}^{\max}\times \left\{1-\exp\left(c\times {\left[CO_{2}\right]}_{j}\right)\right\}\times \left\{1-\exp\left[-Z_{i}\left(T_{j}-T_{LUE}\right)\right]\right\},$$

where $LUE_{i}^{\max}$ is the maximum LUE of a given genotype i, ${\left[CO_{2}\right]}_{j}$ defines the m levels of CO2 concentrations, constant across all growing season, c is a constant, and $\left\{1-\exp\left[-Z_{i}\left(T_{j}-T_{LUE}\right)\right]\right\}$ represents a genotypic-specific exponential reduction in LUE for temperatures below 25 °C with TLUE a temperature level chosen to have the reduction more expressive for temperatures below 20 °C. The leaf area index (LAI) for genotype i in environment j and day t is the product of leaf area per shoot and shoot density and can be written as

(3) $$LAI_{i,j,t}=\left[a+\;B_{i}\left(T_{j}-T_{base}\right)\times \left(t-t_{0}\right)\right]\times Sd,$$

where a and Bi the constant intercept and the genotype specific slope in a regression of the leaf area per stem (m2) as a function of the temperature sum (°C d), Tbase is the base temperature, t represents the t-th day of the growing season (t = t0 is the day of the first flowering), and Sd is the stem density. The photosynthetically active radiation (PAR) incident in the crop (I) on day t in environment j, Ij,t, can be written as

(4) $$I_{j,t}=rad_{j,t}\times F_{PAR}\times Tr_{j}.$$

where radj,t is the global radiation at day t in environment j, FPAR is the fraction of PAR in global radiation, and Trj is the greenhouse transmissivity in environment j. More details about this model can be found in Rodrigues [8] and Rodrigues et al. [7].

The model in Eq. (1) was made specific for sweet pepper. The seven physiological parameters in model (1) that have genotype-specific values will be arranged in the vector P=[LUEmax, K, B, FTF, W, FDMC, Z]. The calibration of the vector P was done based on **a** priori knowledge and assumed to follow a multivariate normal distribution with mean vector

(5) $$\mu^{\prime }=\left[0.87,0.6,0.7,0.000378,0.65,0.04,0.0774\right],$$

and a diagonal variance-covariance matrix

(6) $$V=diag\left(0.174^{2},0.05^{2},0.04^{2},{\left(3.78\times 10^{-5}\right)}^{2},0.04^{2},0.011^{2},0.00508^{2}\right).$$

The remaining constants in the model, for the particular case of sweet pepper, are defined in Table 1.

**Table 1:** Parameterization of the constants in the model defined by Eqs. (1) to (4), for the sweet pepper case (adapted from [8] and from [3]).

Constant	Equation	Value (s)
Tj	Eqn 1	15, 20, 25 (ºC)
TFTF	Eqn 1	15 (ºC)
t0	Eqn 1	January 10 (NL); September 10 (SP)
tf	Eqn 1	November 30 (NL); April 30 (SP)
c	Eqn 2	−0.004
CO2	Eqn 2	370, 1000 (μmol mol-1)
TLUE	Eqn 2	13 (ºC)
a	Eqn 3	0.03372
Tbase	Eqn 3	10 (ºC)
Sd	Eqn 3	7 (per m2)
rad	Eqn 4	Numerical variable
Fpar	Eqn 4	0.5
Tr	Eqn 4	0.75 (NL); 0.60 (SP)

As in Rodrigues et al. [7], 12 environments were defined for two levels of radiation based on available historical data for Spain (SP) and The Netherlands (NL), and three levels of daily average temperature (l = 15, 20 and 25 °C). A time integration of one day was considered for each of the 12 environments.

Each run of the multivariate normal distribution defined by Eqs. (5) and (6), together with the information in Table 1, introduced in model (1) defines one genotype. Running the model 200 times for each of the 12 environments results in a two-way table with 200 rows/genotypes and 12 columns/environments of yield for sweet pepper.

**Fig. 1:**
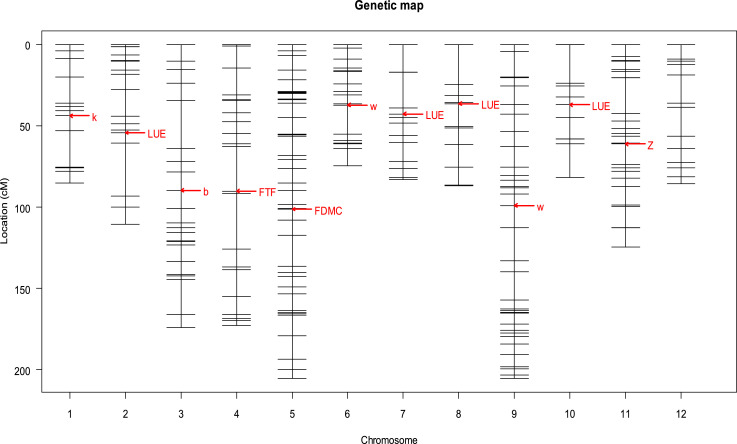
Genetic map for pepper, based on the lengths of chromosome and number of markers per chromosome in Barchi et al. [1]. The marker positions were taken as random. The arrows with the name of the 7 physiological parameters point the place where the QTL were placed.

Following the procedure in Rodrigues [8] and Rodrigues et al. [3], the genetic map (Fig. 1) was simulated based on the lengths of chromosome and number of markers per chromosome in Barchi et al. [1]. A number of QTLs were assigned to the seven physiological parameters as shown in Fig. 1. The simulation was conducted using the function sim.map in package qtl of Software R was used [2], which allowed us to control the level of heritability in the environments.

## Ethics Statement

This work neither involves human subject nor animal experiments.

## CRediT Author Statement

**Paulo Canas Rodrigues:** Methodology, Software, Data curation, Validation, Writing – original draft, Writing – review & editing.

## Declaration of Competing Interest

The author declares that they have no known competing financial interests or personal relationships which have or could be perceived to have influenced the work reported in this article.

## Appendix Supplementary materials

Image, application 1

Image, application 2
